# Cellular cAMP Content and Mitochondrial Profile Define Different Subtypes of Ovarian Cancer Cells

**DOI:** 10.3390/ijms262110474

**Published:** 2025-10-28

**Authors:** Daniela De Benedictis, Aasia Bibi, Luigi Leonardo Palese, Antonella Cormio, Clara Musicco, Vera Loizzi, Gennaro Cormio, Ali Abdelhameed, Domenico De Rasmo, Anna Signorile

**Affiliations:** 1 Department of Translational Biomedicine and Neuroscience, University of Bari Aldo Moro, 70124 Bari, Italy; debenedictis.d0@gmail.com (D.D.B.); aasiabibi250@gmail.com (A.B.); luigileonardo.palese@uniba.it (L.L.P.); vera.loizzi@uniba.it (V.L.); 2 Department of Precision and Regenerative Medicine and Ionian Area, University of Bari Aldo Moro, 70124 Bari, Italy; antonella.cormio@uniba.it; 3 Institute of Biomembranes, Bioenergetics and Molecular Biotechnologies (IBIOM), National Research Council of Italy (CNR), 70124 Bari, Italy; c.musicco@ibiom.cnr.it; 4 S.C. Ginecologia Oncologia Clinicizzata, IRCCS Istituto Tumori Giovanni Paolo II, 70124 Bari, Italy; gennaro.cormio@uniba.it; 5 Department of Interdisciplinary Medicine, University of Bari ‘Aldo Moro’, 70124 Bari, Italy; 6 Department of Pharmaceutical Chemistry, College of Pharmacy, King Saud University, 11451 Riyadh, Saudi Arabia; asaber@ksu.edu.sa

**Keywords:** mitochondria, cAMP, ovarian cancer

## Abstract

Ovarian cancer (OC) is an aggressive and lethal gynecologic cancer due to its asymptomatic nature resulting in a late diagnosis. OC encompasses distinct histological subtypes, with serous OC representing the most common and aggressive form. However, within the same histological OC subtype, additional heterogeneity has been found in terms of genetic mutations and metabolic profiles probably contributing to treatment response. In cancer, metabolic reprogramming strongly involves mitochondria. Mitochondrial function can be regulated by the cAMP pathway, and its deregulation has been reported in various cancers including OC. Here we analyzed two serous OC cell lines, OC316 and OV56, and eleven human OC tissues. OC316 cell lines showed elevated cAMP level with respect to OV56. The high cAMP levels were associated with activation of thecAMP/PKA/CREB/PGC-1α axis resulting in increased mitochondrial biogenesis, respiratory chain activity, modulation of mitochondrial dynamics and apoptosis resistance. Accordingly, principal component analysis (PCA) of the twenty-three biochemical parameters, in eleven human OC tissues, classified OC into two groups showing different cAMP levels associated with distinct mitochondrial profiles. This analysis highlights a cAMP-dependent stratification revealing two mitochondrial subpopulations within serous OC. These findings indicate that the molecular heterogeneity of OC poses a challenge for understanding disease mechanisms and developing effective targeted therapies.

## 1. Introduction

Ovarian cancer (OC) is one of the most aggressive and lethal gynecologic cancers, often diagnosed at advanced stages due to its asymptomatic nature in early phases of disease [1,2]. OC arises from three primary cellular lineages—epithelial, stromal, and germ cells and is classified accordingly based on its tissue of origin [3,4]. The majority of OC cases are epithelial in origin, with the most common subtypes being serous, mucinous, endometrioid, and clear cell carcinoma. This subtype diversity contributes significantly to the overall heterogeneity of OC, influencing disease behavior, therapeutic response and patient outcomes. Among these, high-grade serous ovarian cancer (HGSOC) is the most prevalent and aggressive form, accounting for about 70% of all OC diagnoses [5,6]. At the molecular level, OC exhibits substantial variability in genetic mutations and metabolic profiles [7,8,9]. Notably, mutations in the BRCA1 and BRCA2 genes are frequently observed in HGSOC but occur less commonly in other subtypes [7,8]. Studies have shown that mitochondrial bioenergetics, mitochondrial dynamics, including alterations in mitochondrial structure, number, and oxidative capacity, contribute to OC, affecting the cancer cell’s ability to undergo apoptosis and respond to therapies [10,11,12,13,14,15,16]. Although dysfunctional mitochondria and their structural dysregulation have been associated in general with cancer cells [17,18], a particular heterogeneity has been reported in the serous subtype of OC cells. In fact, bioenergetic analyses have established the existence of a subgroup of OC serous cells, characterized by a high mitochondrial activity, and another subgroup of OC serous cells showing a low mitochondrial activity [19].

In the context of mitochondrial function, cAMP/PKA signaling plays a role in the modulation of mitochondrial function and morphology [20,21,22,23,24] and dysregulation of the cAMP signaling pathway has been reported in various cancers including OC [11,25,26,27]. In particular, in a previous study, we showed that in human OC tissues the levels of cAMP are higher with respect to the control tissues [11]. Moreover, the alteration of cAMP levels in OC has been associated with the alteration of proteins such as SIRT3 and OPA1 [11,12] that strongly contribute to mitochondrial dynamics and apoptosis [24,28]. Furthermore, cAMP signaling is of particular interest in ovarian cancer considering that it is involved, with hormones, in the control of reproductive function [29] and can modulate numerous physiological processes, including gene expression, metabolism, proliferation, differentiation, and cell death [30].

Given the variability in mitochondrial bioenergetics among serous OC cells [19] and the role of cAMP signaling in regulating mitochondrial function [20,21,22,23,24], we investigated cAMP levels, some mitochondrial parameters, and apoptosis resistance in serous OC cell lines and tissues in order to evaluate if the same subtype of tumor exhibits different molecular aspects. Here, we show that cAMP levels and mitochondrial phenotypes, within serous OC, stratified OC into two distinct groups with different molecular profiles and apoptosis responses. We highlight that elucidating the molecular basis of OC heterogeneity represents a significant treatment challenge, as a one-size-fits-all approach is often ineffective.

## 2. Results

### 2.1. cAMP Levels and Cellular Growth Rate in OC316 and OV56 Ovarian Cancer Cells

**Figure 1:**
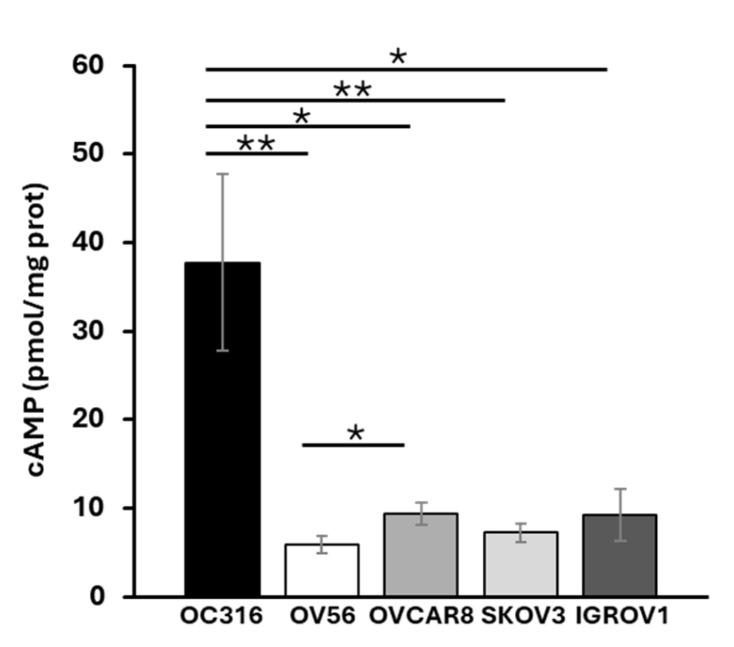
Cellular cAMP levels in different OC cell lines. Total cAMP levels were measured as described in the Materials and Methods section. After collecting in HCl, the cells were centrifuged at 700× *g*. The supernatant was used for direct immunoenzymatic assay. cAMP level was normalized to the protein concentration of the same sample and expressed as pmol/mg of protein. The histograms represent the mean values ± standard error of the mean (SEM) of four independent determinations (*n* = 4) with two technical replicates each. * *p* < 0.05 ** *p* < 0.01 (Student’s *t*-test).

Total cellular cAMP levels were assessed in five ovarian cancer cell lines: OC316, OV56, OVCAR8, SKOV3, and IGROV1. The analyses revealed that, among the examined cell lines, OC316 showed significantly higher cAMP levels (Figure 1). The most significant differences in cAMP levels were observed between OC316 and OV56 cells. Although focusing on only two cell lines, which show the highest and lowest cAMP values, could be a potential limitation, these two cell lines were selected to investigate potential differences in the mitochondrial phenotype in response to cAMP levels.

**Figure 2:**
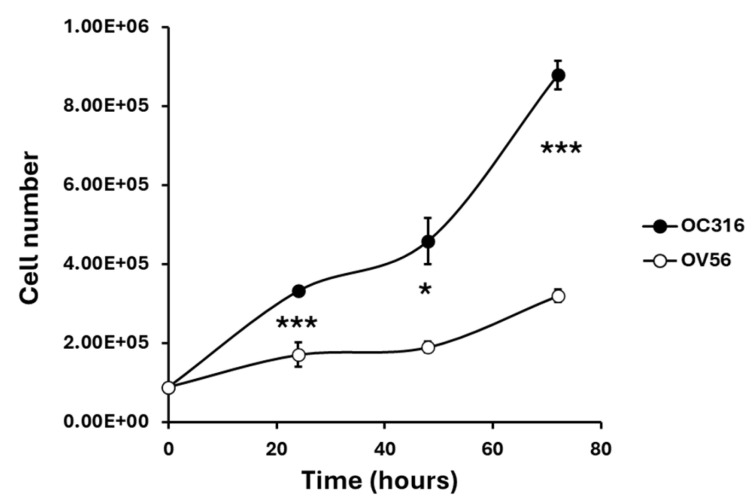
Growth curves of OC316 and OV56 ovarian cancer cells. A total of 90,000 cells were seeded and then counted after 24, 48, and 72 h. The graph represents the mean values ± SEM of three independent determinations (*n* = 3) with three technical replicates each. * *p* < 0.05, *** *p* < 0.001 (Student’s *t*-test).

The two cell lines, grown under the same experimental conditions, showed different growth curves. OC316 cells exhibited significantly higher proliferative activity than OV56 cells, as shown by cell counts performed at 24, 48, and 72 h after seeding an equal number of cells (Figure 2).

### 2.2. Mitochondrial Biogenesis and Function in OC316 and OV56 Ovarian Cancer Cells

It is widely reported that the cAMP signaling cascade plays a crucial role in mitochondrial modulation [21,23,31,32,33,34]. These observations prompted us to investigate, at the molecular level, the effects of different cAMP levels in these two tumor cell lines. One downstream effector of cAMP is the CREB protein, a transcription factor that, once phosphorylated by PKA, increases the expression of target genes involved in mitochondrial homeostasis, cell migration, proliferation, and apoptosis [35].

**Figure 3:**
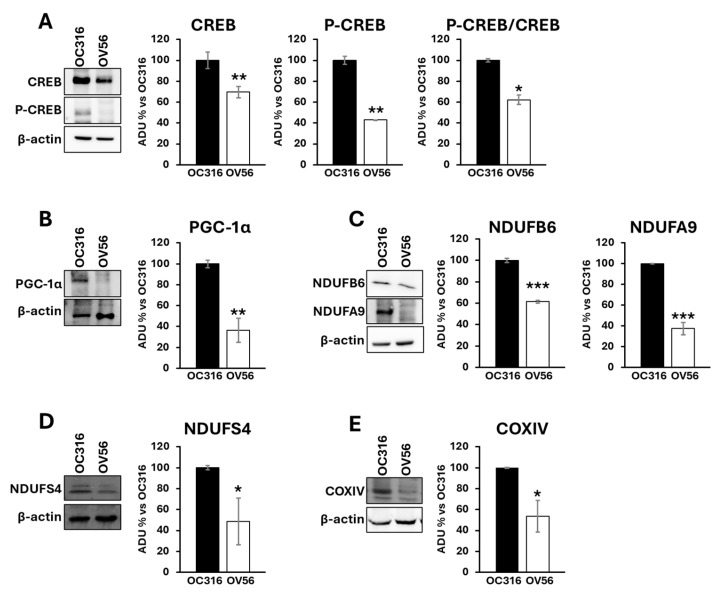
Expression and phosphorylation of CREB protein, PGC-1α and subunits of mitochondrial respiratory chain complexes in OC316 and OV56 cells. Proteins from cellular lysates of OC316 and OV56 were loaded in a 10% SDS-PAGE and transferred to nitrocellulose. The panels show representative images of Western blot using anti CREB and P-CREB (**A**), PGC-1α (**B**), NDUFA9 and NDUFB6 (**C**), NDUFS4 (**D**) and COXIV (**E**). Data in the histograms represent the quantification as arbitrary densitometric units (ADUs) normalized to β-actin levels and expressed as percentage vs. OC316 values. The histogram represents mean values ± SEM of three independent determinations (*n* = 3). * *p* < 0.05; ** *p*< 0,01; *** *p* < 0.001 (Student’s *t*-test).

Proteins from cellular lysates were subjected to electrophoresis and Western blotting using antibodies against CREB and phosphorylated CREB (P-CREB). The analysis revealed a higher abundance of the CREB protein level in the OC316 compared with the OV56 cell line. According to the cAMP levels, the amount of phosphorylated CREB was higher in OC316 cells than in OV56 (Figure 3A). The P-CREB/CREB ratio (OC316 ratio: 4.57 ± 0.29; OV56 ratio: 2.83 ± 0.04) was significantly elevated in OC316 cells with respect to OV56 (Figure 3A). Additionally, Western blotting analysis detected higher PGC-1α levels in OC316 cells compared with OV56 (Figure 3B), as well as higher levels of some subunits of the mitochondrial respiratory chain complexes, such as NDUFA9, NDUFB6, and NDUFS4 of complex I and COXIV of complex IV (Figure 3C–E). These data suggested that the higher cAMP levels were associated with higher CREB and P-CREB levels, as well as higher levels of mitochondrial respiratory chain subunits. This indicates a possible hyperactivation of the cAMP/CREB/PGC-1α pathway, resulting in augmented mitochondrial biogenesis.

**Figure 4:**
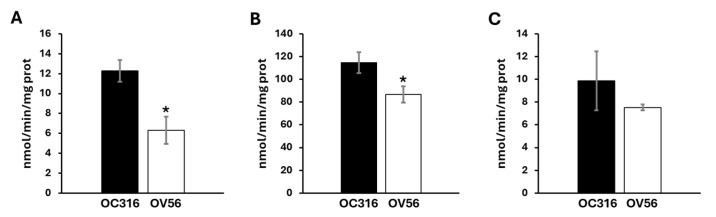
Activity of mitochondrial respiratory chain complexes in OC316 and OV56. (**A**) The activity of complex I was measured spectrophotometrically following the oxidation of NADH at 340 nm. (**B**) The activity of complex III was measured following the reduction of oxidized cytochrome *c* at 550 nm. (**C**) The activity of complex IV was measured following the oxidation of reduced cytochrome *c* at 550 nm. The graphs represent the mean values of Vmax ± SEM of four independent determinations (*n* = 4) with three technical replicates each. * *p* < 0.05 (Student’s *t*-test).

Considering the role of cAMP/PKA signal on mitochondrial respiratory chain activity and the different protein levels of respiratory chain subunits, the enzymatic Vmax activities of the complexes and metabolic flux were analyzed. As shown in Figure 4, OC316 cells showed a higher activity of complex I and complex III compared with the OV56 cell line, while the activity of complex IV in OC316, even if tendentially higher, was not statistically significant.

**Figure 5:**
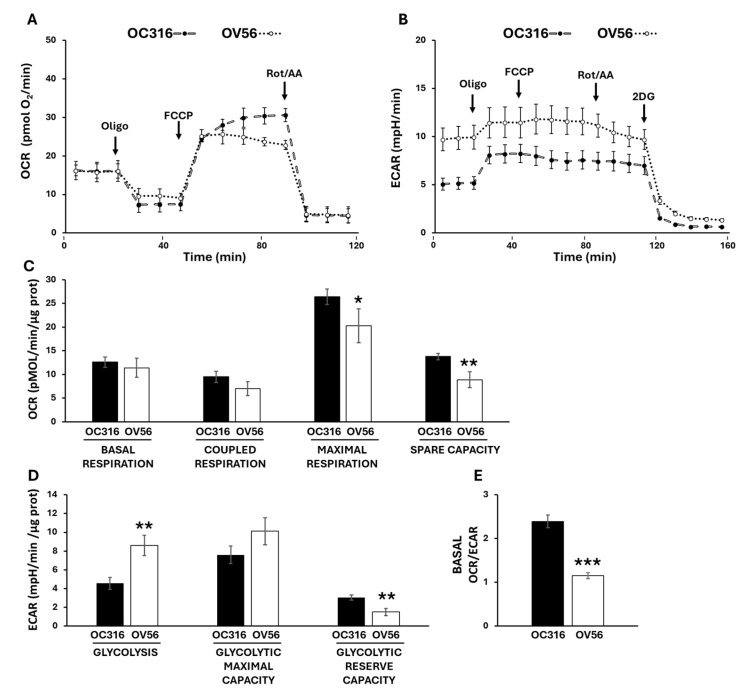
Bioenergetic analysis in OC316 and OV56 cells. Representative profile of Oxygen Consumption Rate (OCR) (**A**) and Extracellular Acidification Rate (ECAR) (**B**) of OC316 and OV56 cells, recorded at baseline and after injection of ATP synthase inhibitor oligomycin (oligo), uncoupler FCCP, complex I inhibitor rotenone (Rot) plus complex III inhibitor antimycin A (AA) and 2-deoxy glucose (2DG). Each data point is the mean  ±  SD of five technical replicates. (**C**) Histograms of metabolic parameters obtained from OCR assay derived for basal respiration, coupled respiration, maximal respiration and spare respiratory capacity in OC316 and OV56 cells. (**D**) Histograms of metabolic parameters obtained from ECAR assay derived for glycolysis, glycolytic maximal capacity and glycolytic reserve capacity in OC316 and OV56 cells. (**E**) Ratio between basal OCR and ECAR. Data in histograms are the mean  ±  SD of three independent experiments with five technical replicates each. * *p*  <  0.05, ** *p*  <  0.01, *** *p*  <  0.001 (Student’s *t*-test). See under “Materials and Methods” for further details.

To investigate cellular bioenergetics we used Seahorse technology to assess the two main metabolic fluxes in intact cells: the mitochondrial oxidative phosphorylation activity, measured as oxygen consumption rate (OCR) (Figure 5A), and the extracellular acidification rate (ECAR) (Figure 5B) mainly contributed by the conversion of pyruvate to lactate, essentially according to the manufacturer’s Mito Stress protocol [36,37]. The histograms show the data quantification obtained by comparing the OC316 and OV56 cells’ OCR (Figure 5C) and ECAR (Figure 5D) activities. The basal respiration, which shows the energetic demand of the cell under baseline conditions, did not change between OC316 and OV56 cells as well as the coupled respiration, that represents how much oxygen consumption is directly linked to ATP synthesis (Figure 5C). The mitochondrial maximal respiration activity, indicating the potential full capacity of the cells, is higher in OC316 cells compared to OV56 cells (Figure 5C). The spare capacity, representing an increased ATP production via mitochondria to respond to stress or increased metabolic demands, is higher in OC316 cells compared with OV56 cells (Figure 5C). On the contrary, the glycolytic activity was higher in OV56 cells as shown by ECAR (Figure 5D). Instead, the glycolytic reserve capacity, the maximal rate at which a cell can increase its ATP production via glycolysis, appeared to be higher in OV56 with respect to OC316 cells (Figure 5D). The OCR/ECAR ratio, higher in OC316 than OV56 cells, suggested a greater reliance on mitochondrial oxidative phosphorylation in OC316 cells (Figure 5E).

### 2.3. Proteins Involved in Mitochondrial Dynamics in OC316 and OV56 Ovarian Cancer Cells

**Figure 6:**
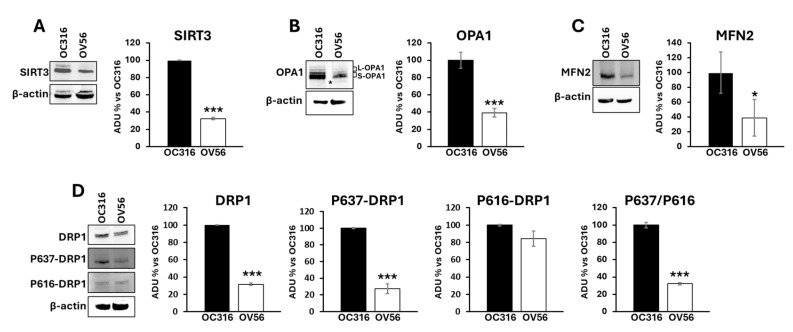
Analysis of mitochondrial-shaping proteins in OC316 and OV56. Proteins from OC316 and OV56 cell lysates were subjected to SDS-PAGE and transferred to nitrocellulose. (**A**) Representative images of Western blotting against SIRT3 (**A**), OPA1 (**B**), MFN2 (**C**), DRP1 and its phosphorylated forms (**D**). The asterisk in panel B represents the faint band of OPA1. Data in the histograms represent the quantification as arbitrary densitometric units (ADUs) normalized to β-actin levels and expressed as a percentage vs. OC316 values. The histograms represent the mean values ± SEM of three independent determinations (*n* = 3). * *p* < 0.05; *** *p* < 0.001 (Student’s *t*-test).

It has been shown that the cAMP/PKA pathway impinges mitochondrial dynamics by modulating SIRT3 protein levels [24,38] and the phosphorylation of DRP1 at serine 637 [39]. SIRT3 is a deacetylase localized in mitochondria and plays an important role in modulating mitochondrial dynamics by regulating the acetylation state of OPA1. OPA1 presents a long (L-OPA1) and a short (S-OPA1) form with the latter generated by proteolytic processing of L-OPA1 [40]. In response to an apoptotic stimulus, the balance between long and short forms changes in favor of S-OPA1 resulting in mitochondrial fragmentation and apoptosis [41,42]. OPA1, once deacetylated by SIRT3, is preserved from the proteolytic process [43]. The cAMP-dependent phosphorylation of DRP1 at serine 637 limits DRP1 in the cytosol inhibiting its activity in mitochondria as a pro-fission protein [39]. For this reason, the levels of mitochondrial shaping-proteins involved in mitochondrial dynamics such as SIRT3, OPA1, mitofusin 2 (MFN2) and DRP1 have been assessed. Figure 6 shows that SIRT3 (panel A) and OPA1 (panel B) protein levels were significantly higher in OC316 cells compared with OV56. Moreover, the proteolytic processing of OPA1 is different with the appearance of a faint band (asterisked in Figure 6B) of short OPA1 forms in OV56 cells (Figure 6B). Also the level of MFN2 was higher in OC316 cells (Figure 6C). Western blotting analysis using antibodies against DRP1 highlighted an increase in DRP1 protein level in OC316 cells with respect to OV56 (Figure 6D). Furthermore, the antibody against the cAMP/PKA-dependent phosphorylation of DRP1 at serine 637 (P637-DRP1) showed a higher phosphorylation in OC316 cells compared with OV56 cells (Figure 6D) while no difference was observed in the phosphorylation form of DRP1 at serine 616 (Figure 6D). Accordingly, the ratio between P637-DRP1/P616- DRP1 (OC316 ratio: 5.51 ± 3.16; OV56 ratio: 1.51 ± 0.74) was higher in OC316 cells, as show in percentage in the figure, indicating a more abundant cAMP-dependent phosphorylation of DRP1.

### 2.4. DAPI Analysis in OC316 and OV56 Ovarian Cancer Cells

**Figure 7:**
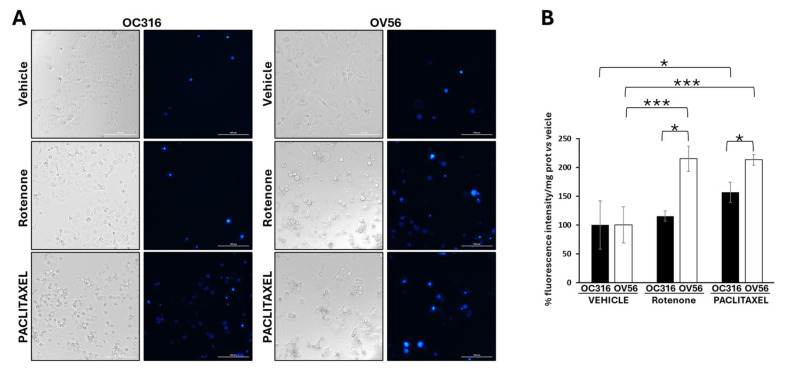
Analysis of DAPI staining in cell lines treated with rotenone and paclitaxel. (**A**) Representative images obtained by Cytation5 Cell Imaging system at 20x magnification of OC316 and OV56 treated for 24 h with DMSO (vehicle), 0.5 µM rotenone or 0.5 µM paclitaxel. (**B**) Data in the histograms represent the quantification of intensity of DAPI fluorescence expressed as percentage vs. OC316 and OV56 values. The histogram represents the mean values ± SEM of three independent determinations (*n* = 3) with three technical replicates each. * *p* < 0.05; *** *p* < 0.001 (Student’s *t*-test).

Considering the key role of cAMP signaling and mitochondria in cellular response to apoptosis [44,45,46,47,48], we performed DAPI analysis in the OC316 and OV56 cell lines in the presence of rotenone, a mitochondrial complex I inhibitor usually used to induce apoptosis [49], or paclitaxel, a chemotherapeutic agent. DAPI can pass through cell membranes, but its uptake is increased in apoptotic cells with compromised membranes, and the overall blue fluorescence intensity of the nucleus increases due to greater DAPI uptake. The acquisition of fluorescent images revealed that vehicle-treated cell lines presented a low level of DAPI staining (Figure 7A). The treatment of OC316 cells with rotenone did not induce an increase in DAPI staining that was, instead, increased by paclitaxel treatment (Figure 7B). The treatment of OV56 cells with rotenone and paclitaxel induced a marked increase in DAPI staining (Figure 7A,B), indicating induction of apoptosis by these agents. Moreover, in both rotenone and paclitaxel treatments, the increase in DAPI staining was statistically significantly higher in OV56 than OC316 cell lines. The bright field microscopy images showed the same quantity of OC316 and OV56 cells after 24 h treatment with the vehicle. An increase in rounded cells after the treatment for 24 h with rotenone or paclitaxel was observed (Figure 7A), suggesting an induction of apoptosis. These data suggested a greater apoptotic resistance to rotenone and paclitaxel of OC316 respect to OV56. 

### 2.5. Effect of H89, Inhibitor of PKA, on the Growth Curve and Apoptosis in OC316 and OV56 Ovarian Cancer Cells

To further verify the involvement of the cAMP/PKA pathway, H89, an inhibitor of PKA activity, was used to evaluate the growth rates and the response to apoptosis stimuli.

**Figure 8:**
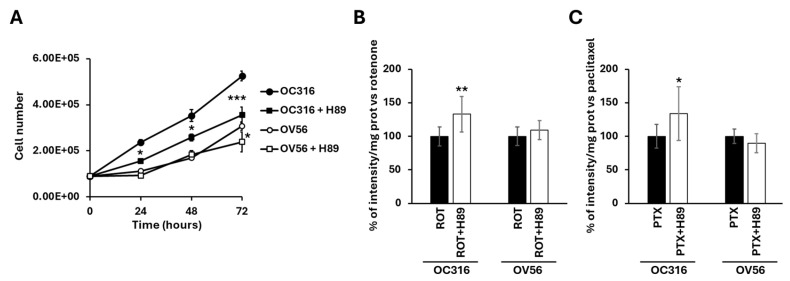
(**A**) Growth curves of OC316 and OV56 ovarian cancer cells. A total of 90,000 cells were seeded in the absence and in the presence of 48 nM H89 and then counted after 24, 48, and 72 h. The graph represents the mean values ± SEM of three independent determinations (*n* = 3) with three technical replicates each. * *p* < 0.05: OC316 vs. OC316 + H89 at 24 and 48 h, OV56 vs. OV56 + H89 at 72 h. *** *p* < 0.001: OC316 vs. OC316 + H89 at 72 h (Student’s *t*-test). In (**B**,**C**) OC316 and OV56 were treated in the absence or presence of 48 nm H89. After 12 h treatments, 0.5 µM rotenone (ROT) or 0.5 µM paclitaxel (PTX) was added for 24 h and DAPI staining was analyzed. Data in the histogram represent the quantification of intensity of fluorescence expressed as a percentage of fluorescence intensity vs. rotenone or paclitaxel-treated OC316 and OV56 values. The histogram represents the mean values ± standard deviation (SD) of three independent determinations (*n* = 3) with three technical replicates each. * *p* < 0.05; ** *p* < 0.01 (Student’s *t*-test).

Figure 8A shows that H89, at the 48 nM concentration, which selectively inhibits PKA, slowed down the growth of O316 cells after 24 h of treatment compared with untreated cells (Figure 8A). In OV56 cell line the growth was significantly inhibited at 72 h of H89 treatment (Figure 8A).

For apoptosis, cells were treated for 24 h with H89 before the drug-dependent apoptosis induction. As shown in Figure 7A, bright-field microscopy indicated that rotenone treatment resulted in an increase in rounded OC316 cells, suggesting induction of OC316 cell apoptosis by rotenone. In these cells, induction of apoptosis was low, which was determined by comparing the bright-field images of OC316 cells treated with rotenone with those of OC316 cells treated with paclitaxel and with OV56 cells treated with rotenone and with paclitaxel (Figure 7A), resulting in no overt increase in DAPI staining of the rotenone-treated OC316 cells (Figure 7B). However, DAPI staining of rotenone-treated OC316 cells pre-treated with H89 indicated that H89 was able to increase the apoptosis induced by rotenone (Figure 8B). H89 also increased the apoptosis induced by paclitaxel (Figure 8C). In the OV56 cell line, the H89 did not significantly increase the DAPI staining, probably due to a lower PKA activity (Figure 7B,C). In agreement with these results, the MTT test (3-(4,5-dimethylthiazol-2-yl)-2,5-diphenyltetrazolium bromide) (Figure S1) showed that the pre-treatment with H89 slightly reduced the percentage of live cells only in rotenone or paclitaxel-treated OC316 cells. 

### 2.6. Analysis of Ex-Vivo Human Tissues Derived from Serous OC

**Figure 9:**
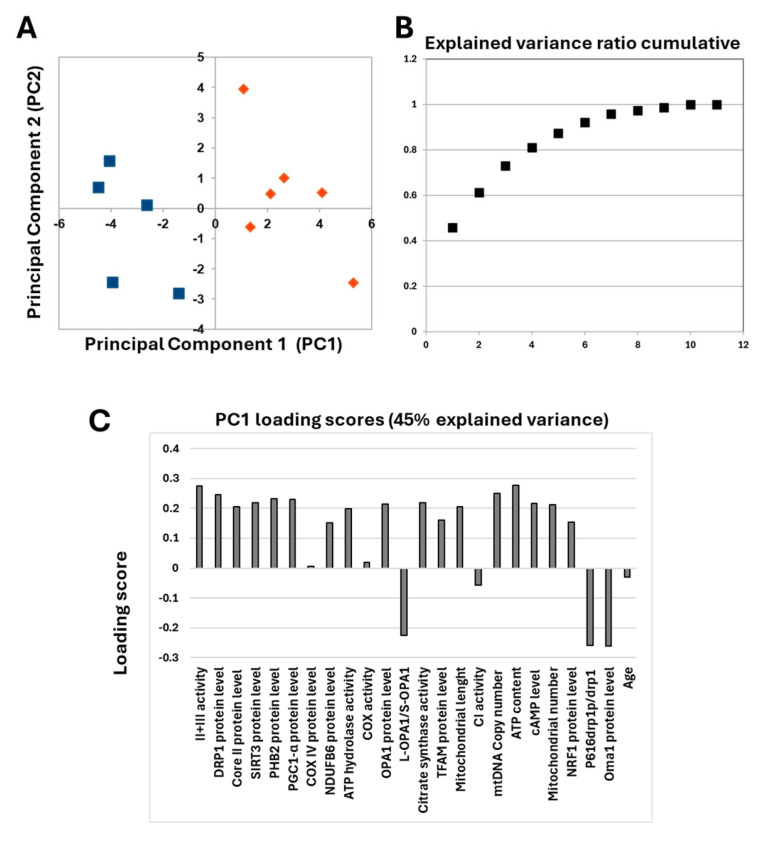
(**A**) Principal component analysis. The figure reports the projection onto the first two principal components of the analyzed dataset. The two populations are represented by blue squares and orange diamonds. For the analysis, the following parameters were used: cAMP levels, complex I activity, complex IV activity, complex II + III activity, ATP hydrolase activity, citrate synthase activity, NDUFB6 protein level, Cox IV protein level, core II protein level, ATP content, TFAM protein level, PGC1-α protein level, NRF1 protein level, mtDNA copy number, mitochondrial number, SIRT3 protein level, PHB2 protein level, OPA1 protein level, L-OPA1/S-OPA1 ratio, OMA1 protein level, DRP1 protein level, P616-DRP1/DRP1 ratio, mitochondrial length and age. (**B**) Explained variance ratio cumulative. (**C**) Loading scores values of all parameters.

The obtained data showed that different levels of cAMP in the two serous OC cell lines were associated with characteristic mitochondrial profiles. This prompted us to investigate ex vivo human tissues derived from serous OC biopsies. Already published data on serous OC tissues have shown a marked increase in cAMP levels compared with control sample tissues, which is associated with a specific mitochondrial signature characterized by an increase in mitochondrial biogenesis and cristae remodeling, suggesting increased resistance to apoptosis in OC cells [11]. The re-evaluation of the data from only serous OC tissues by PCA analysis revealed that these serous OC tissues separated into two distinct populations in the first dimension (Figure 9A). 

In addition, the explained variance indicated that more than 45% of the total variance is explained with just one component (Figure 9B). The analysis of PC1 loading scores showed that mitochondrial function parameters, as well as cAMP levels, are all important for the linear separation observed between the two populations with slight scores for NDUFB6, COREII, TFAM and NRF1 protein levels. The only parameters that did not seem to correlate with the presence of two different populations are those related to the content and activity of cytochrome c oxidase, complex I activity and age. These data prompted us to perform a detailed statistical analysis of mitochondrial parameters in the two populations.

As reported in Table 1 the two OC populations differed significantly in cAMP levels and in several markers of mitochondrial bioenergetics (complex II-III activity, ATP content and citrate synthase activity), mitochondrial biogenesis (PGC-1α protein level, mitochondrial DNA copy number and number of mitochondria), and mitochondrial dynamics (SIRT3, prohibitin 2 (PHB), DRP1, P616-DRP1 ratio, OPA1 protein levels, and mitochondrial length) (see Figure S2 for Western blotting images). Therefore, the two OC populations have been marked as H-OC and L-OC because, respectively, they showed a higher (H) and a lower levels (L) of cAMP (Table 1).

## 3. Discussion

OC is one of the most aggressive and fatal gynecological cancers, primarily due to its asymptomatic nature and, thus, a late diagnosis [1]. OC can be classified into several histological subtypes according to the cell of origin [3], including serous, mucinous, endometrioid, and clear cell carcinomas [5]. The heterogeneity of OC is not only confined to the histological subtypes but it extends to genetic, molecular and metabolic profiles [6,50,51] thus, probably, resulting in different responses of patients to the therapy. However, the specific genetic profiles described for OC cell lines do not match the metabolic profiles. For example, in the paper by Domcke et al. [52], the cell lines Kumarachi, OVCAR8 and IGROV1 were genetically classified as likely high-grade serous, possibly high-grade serous and hypermutated, respectively. Instead, these cell lines are grouped in the same metabolic bioenergetic profile in another work [19]. This highlights the complexity in the classifications of OC that can involve histological, genetic and molecular aspects.

Here we add a further point of complexity showing that, although cAMP levels are generally elevated in serous OC compared with controls [11], at the molecular level, within the same subtype of OC (serous), cAMP levels can vary significantly and it is associated with a characteristic mitochondrial profile.

The initiation and progression of tumors are often associated with the dysregulation of signaling pathways, and numerous protein kinases and phosphatases have emerged as potential therapeutic targets [53,54]. cAMP signaling regulates a wide range of cellular processes, including gene expression, metabolism, proliferation, differentiation, and apoptosis [30,55]. Dysregulation of this pathway has been implicated in metabolic, neurodegenerative, and proliferative diseases [26,56,57]. Notably, the cAMP/PKA signaling cascade can influence both mitochondrial structure and function [20,23,24,33,39,58,59,60]. Based on these premises, total cAMP levels were assessed in eleven serous OC tissues and in two serous OC cell lines. Analysis conducted in OC tissues reveals the presence of two subpopulations of OC that showed significant differences in cAMP levels. We have defined these subgroups as H-OC (cAMP values 9.33 pmol/mg prot ± 3.33) and L-OC (cAMP values 2.96 pmol/mg prot ± 1.12). Analysis of cAMP levels in OC cell lines revealed different cAMP contents, in particular, OC316 showed a higher cAMP level with respect to OV56. In OC316 cell line, higher cAMP levels were also associated with greater cell growth capacity [61,62]. Of note, the growth rate is inhibited by the addition of H89, an inhibitor of PKA. However, even if at the used concentration (48 nM) H89 should inhibit only the PKA, the contribution of other kinases and of off-target effects in growth capacity cannot be completely ruled out [63]. In agreement with the growth cell capacity, it has been shown that subcutaneous xenografts derived from OC316 cells showed a doubling time of a few days, indicating a high proliferation rate [64]. In addition, the mice inoculated with the subcutaneous OC316 cell line developed an extremely aggressive and cisplatin-resistant tumor [65]. On the contrary, xenograft tumor growth of OV56 cells showed poor in vivo growth [66]. It should be mentioned that OV56 is a cell line that does not naturally express MAGE-A11 (melanoma-associated antigen 11). MAGE-A11 is a protein aberrantly expressed in many tumors acting as an oncogene [67,68,69,70]. It has been found that the stable overexpression of MAGE-A11 in OV56 significantly increased the tumor growth in mice [66] and, of note, MAGE-A11 expression is controlled by cAMP [67].

A key downstream effector of cAMP signaling is the transcription factor CREB, which regulates genes involved in mitochondrial biogenesis, migration, proliferation, and apoptosis [35]. After PKA activation, mediated by high cellular cAMP levels, its catalytic subunits translocate to the nucleus where it phosphorylates CREB [71]. Phosphorylated CREB also influences PGC-1α expression. PGC-1α is a transcriptional coactivator that regulates numerous aspects of cellular metabolism and represents a “master gene” for mitochondrial biogenesis [72,73]. PGC-1α is a transcriptional coactivator that acts by triggering other nuclear transcription factors, such as NRF-1 and NRF-2, which in turn promote the expression of genes essential for mitochondrial function and biogenesis, including TFAM, which is critical for mitochondrial DNA replication and transcription [72,73,74]. 

Consistent with this pathway, higher cAMP levels were associated with an increase in phosphorylation of the CREB protein (suggesting an activation of PKA), PGC-1α protein levels, respiratory complexes activities, citrate synthase activity and respiratory chain subunit levels in both OC316 cells and H-OC tissues. Moreover, metabolic flux analysis confirmed that OC316 cells, characterized by high cAMP, relied more heavily on mitochondrial metabolism compared with OV56 cells. It is noteworthy that CREB has been shown to suppress or activate apoptosis in OC. While it is reported that the hyperactivation of the cAMP/CREB pathway [75], as well as a greater mitochondrial activity [19], results in a loss of chemotherapy resistance in OC, the inhibition of CREB phosphorylation has been shown to sensitize tumor cells to platinum-based therapy, thereby limiting cancer recurrence [27,76]. This duality emphasizes the complexity of OC and highlights the need for integrated molecular and metabolic profiling.

Mitochondria are dynamic organelles that fuse (fusion) and divide (fission) continuously, adjusting their shape in response to cellular energy demands [77]. At the same time, the dynamic changes in mitochondrial shape affect cell survival and cell death and are important in drug resistance [18]. Several proteins are involved in the dynamics of mitochondria, including SIRT3, MFN2 and OPA1, for mitochondrial fusion, and DRP1 for mitochondrial fission [24,77]. In both OC cell lines and human OC tissues, the increase in cAMP levels is also associated with an increase in SIRT3 protein levels. Indeed, the protein level of SIRT3, the main mitochondrial deacetylase, can be mediated by cAMP levels, as demonstrated by the fact that a decrease in mitochondrial cAMP levels can activate mitochondrial proteases that induce a proteolytic-dependent decrease in SIRT3 protein levels. In turn, a low level of SIRT3 causes a decrease in the deacetylation of its substrate OPA1. Hyperacetylated OPA1 is subject to proteolytic processing generating short isoforms of OPA1 inducing mitochondrial fragmentation (fission) that makes cells more susceptible towards apoptosis [24,41]. SIRT3 has emerged as a key regulator of tumor cell metabolism, its role can be either pro-oncogenic or tumor suppressive depending on the cell type [78,79,80,81,82]. In OC cells the role of SIRT3 is controversial, in fact in the OC SKOV3 cell line, the overexpression of SIRT3 can induce cell death but it has also been found that SIRT3 activation has been associated with an increase in OC sensitivity to cisplatin [83]. This double aspect of SIRT3, again can depend on the entire molecular and metabolic profile.

OPA1, in addition to its fundamental role in mitochondrial fusion, can regulate cristae structure and resistance to apoptosis [84]. L-OPA1 processing is also mediated by OMA1 protease [40,42] that, which we found to be lower in H-OC tissues compared with respect to L-OC tissues. According to cAMP, SIRT3, and OMA1 protein levels, we found an in-creased L-OPA1/S-OPA1 ratio, in H-OC tissues. On the other hand, a higher level of cAMP in OC316 cells was associated with a different processing of OPA1.

Other proteins involved in the mitochondrial fusion process are represented by mitofusins (MFN1/2). MFN2 is one of the most important mediators of mitochondrial fusion and mitochondria-endoplasmic reticulum interaction and mitophagy [77,85]. Many data indicated that MFN2 alterations are associated with mitochondrial dysfunction, which can influence tumor onset and progression. Altered expression of MFN2 has been, for example, reported in different types of tumors such as hepatocellular carcinoma, breast, lung, cervical and pancreatic cancer [86,87,88]. We found an augmented MFN2 protein levels in the OC cell line and OC tissues with higher cAMP levels.

Mitochondrial dynamics is also regulated by fission proteins. The most important protein in the fission process is represented by DRP1 [77]. The involvement of DRP1 in the fission process is regulated by the phosphorylation of two serines. The cAMP/PKA-dependent phosphorylation of serine 637 inhibits the translocation of DRP1 from cytosol to mitochondria avoiding the pro-fission activity of DRP1 in mitochondria and therefore represents a pro-fusion element [39]. On the contrary, DRP1 phosphorylation of serine 616 is a pro-fission event [89]. Western blotting analysis using antibodies against DRP1 highlighted an increase in DRP1 protein levels in both the OC316 cell line and H-OC tissues. Moreover, in OC316, an increase in the P637-DRP1 was associated with increased cAMP levels, and in H-OC tissues, a decrease in P616-DRP1 was associated with increased cAMP levels. Finally, our experiments of paclitaxel-induced apoptosis in cell lines indicated a great resistance towards apoptosis in OC316 compared with OV56 cells, associated with higher cAMP levels and a characteristic molecular and metabolic mitochondrial profile. All these data, involving cAMP-dependent regulation of mitochondria, revealed two distinct molecular mitochondrial phenotypes in the same kind of OC. Due to the limited tissues and cell lines examined, other studies are needed to confirm our observations and to evaluate the patient outcome or chemotherapy response. However, even if further studies are needed, these phenotypes could be associated with changes in mitochondrial biogenesis and fusion processes influencing the propensity for apoptosis, offering new interesting challenges.

## 4. Materials and Methods

### 4.1. Cell Cultures

OC316, OV56, OVCAR8, IGROV1 e, and SKOV3 cell lines were kindly donated by Riccardo Spizzo (CRO Aviano, Aviano PN, Italy) (for details see Supplementary Table S1). Cells were grown in DMEM supplemented with 10% South American fetal bovine serum (FBS) (Euroclone, Pero, Italy), 1 mM sodium pyruvate (Euroclone) plus 4 mM glutamine, and 100 IU/mL penicillin and 100 IU/mL streptomycin at 37 °C, in a humidified atmosphere of 5% CO_2_. Once at 80–90% confluence, cells were harvested and used for the analysis. Further specifications are provided in the legends to the figures. For the growth curve 90.000 OC316 or OV56 cells were seeded in 10 mm culture dishes (Falcon, Primaria Easy Grip; Becton Dickinson, Franklin Lakes, NJ, USA). Cells were detached using trypsin and counted using a Scepter cell counter (Merck Millipore, Burlington, MA, USA) at 24, 48, and 72 h post-seeding.

### 4.2. cAMP Assay

Cells were collected by adding 500 µL of 0.1 M HCl in the presence of 0.1% Triton X-100 for 15 min at 37 °C, followed by manual scraping. The lysate was then centrifuged at 700× *g* for 5 min at 4 °C and the supernatant was recovered. cAMP levels were measured using the Direct cAMP ELISA Kit (Enzo Life Sciences, New York, NY, USA) according to the manufacturer’s protocol. Absorbance was measured with the Cytation5 Cell Imaging Multi-Mode Reader (BioTek, Winooski, VT, USA). The cAMP values were normalized to the protein concentration and expressed as pmol/mg.

### 4.3. Electrophoretic Procedures and Western Blotting

The cells were harvested and pelleted by centrifugation at 600× *g* and then resuspended in PBS pH 7.4 in the presence of a protease inhibitor (0.25 mM PMSF). The cellular proteins were resuspended in lysis buffer and separated by 10% SDS-polyacrylamide gel electrophoresis (PAGE) and transferred to a nitrocellulose membrane. The nitrocellulose membrane was blocked with 5% fatty acid free dry milk in 20 mM Tris, 500 mM NaCl, and 0.05% Tween 20, at pH 7.4 (TTBS) for 2 h at 4 °C and probed with antibodies described in the legend to figures. Densitometric analysis was performed by Image Lab software 2.1 (BioRad, Milan, Italy).

### 4.4. Enzymatic Activities

The whole-cell lysates were exposed to ultrasound energy for 15 s at 0 °C.

The NADH-UQ oxidoreductase activity (complex I) was performed in 40 mM potassium phosphate buffer, pH 7.4, and 5 mMMgCl_2_, in the presence of 3 mM KCN, 1 μg/mL antimycin, 200 μM decylubiquinone, using 50 μg of mitoplast proteins, by following the oxidation of 100 μM NADH at 340–425 nm (Δε = 6.81 mM^−1^ cm^−1^). The activity was corrected for the residual activity measured in the presence of 1 μg/mL rotenone.

Cytochrome c oxidase (complex IV) activity was measured by following the oxidation of 10 μM ferrocytochrome c at 550–540 nm (Δε = 19.1 mM^−1^ cm^−1^). Enzymatic activity was measured in 10 mM phosphate buffer, at pH 7.4, using 20 μg of mitoplast proteins. This rate was inhibited by over 95% by KCN (2 mM).

Ubiquinol-cytochrome *c* oxidoreductase (complex III) activity was measured at 550–540 nm (Δε = 19.1 mM^−1^ cm^−1^) as the initial rate of antimycin-sensitive cytochrome c reduction.

### 4.5. Metabolic Flux Analysis

Oxygen consumption rate (OCR) and extracellular acidification rate (ECAR) were measured as key bioenergetic parameters of OC316 and OV56 cells using the Agilent Seahorse XFe24 analyzer (Agilent Technologies, Santa Clara, CA, USA) and XF cell Mito Stress Test (Agilent) following the manufacturer’s instructions.

Briefly, after optimization of the seeding density, 1.5 × 10^4^ cells were seeded in XFe24 plates and incubated until complete adhesion in XF RPMI medium supplemented with 10 mM glucose, 2 mM L-glutamine and 1 mM sodium pyruvate in a non-CO_2_ incubator at 37 °C. OCR and ECAR were measured at intervals by mixing and waiting periods, using a set number of replicates per experimental point: basal OCR measurements were followed by oligomycin injection (1 µM), to inhibit ATP synthase, followed by sequential injections of FCCP (1.5 μM), to uncouple mitochondrial respiration, and rotenone  +  antimycin A (1 µM  +  1 µM), to inhibit mitochondrial respiration. For ECAR analysis, at the end of running, 50 mM 2-deoxy-d-glucose was injected to shut down glycolysis and allow data correction for non-glycolytic medium acidification.

Mitochondrial parameters were calculated as an average of five technical replicates as follows: basal respiration corresponds to the difference between OCR measurement and OCR plus mitochondrial respiratory chain inhibitors; coupled respiration is the difference between basal respiration and OCR measurement after oligomycin injection; maximal respiration is the difference between OCR plus FCCP and OCR plus mitochondrial respiratory chain inhibitors; and spare capacity corresponds to the difference between OCR plus FCCP and basal OCR.

ECAR parameters were calculated as an average of five technical replicates as follows: glycolysis is the difference between the basal measurement and the measurement after 2-deoxyglucose injection; glycolytic maximal capacity corresponds to the difference between ECAR plus oligomycin and ECAR plus 2-deoxyglucose injections; and glycolytic reserve capacity is calculated as the difference between ECAR plus oligomycin and ECAR basal measurement. The OCR and ECAR values were normalized to protein content in each well, determined by the Pierce BCA Protein Assay Kit (Thermo Fisher Scientific, Waltham, MA, USA).

### 4.6. Diamidino-2-phenylinole (DAPI) Staining

For DAPI staining, OC316 and OV56 cell lines were grown onto Primo flat bottom 96 wells plates (EuroClone). Cells were washed with PBS and incubated with 0.3 μM DAPI (Thermo Fisher Scientific) for 5 min at 37 °C. Images were acquired with a 20 × objective lens (excitation 377 nm; emission 447 nm) and fluorescence intensity reading was performed at 450/50 nm in a scanning mode (7 measurements/well) using a Cytation5 Cell Imaging Multi-Mode Reader (BioTek). Fluorescence intensity data of each well were normalized to the protein concentration, determined using a Pierce BCA Protein Assay Kit (Thermo Fisher Scientific).

### 4.7. Patient Samples and Analysis

The study was conducted on 11 subjects with ovarian cancer (histological subtypes serous grade G3). The OC tissue samples were taken during surgery to remove the tumor mass. Patients were selected by the Department of Gynecology at the University of Bari Policlinico. Tissue samples were taken and frozen at −80 °C and none of the patients received any treatment (radiotherapy, chemotherapy, or hormone therapy) before surgery. The study was approved by the Independent Ethical Committee—IEC—Azienda Ospedale Consorziale Policlinico, Bari (n. 3574, 29 November 2017), and the consent form for participation was distributed to all participants and signed. All used methods were performed in accordance with the ethical standards of the institutional and national research committee and with the 1964 Helsinki declaration and its later amendments or comparable ethical standards. All the analyses were performed as described in [11] except for Western blotting for OMA1, NRF1, NDUFB6, Core II, DRP1, P616-DRP1 and P637-DRP1 proteins. In brief, the sample tissues (100–400 mg) were homogenized in 0.25 M mannitol, and 10 mM Tris plus 0.25 mM phenylmethylsulfonyl fluoride (PMSF) and subjected to ultrasound treatment. The proteins were, then, separated in 8% SDS-PAGE, transferred to a nitrocellulose membrane and blocked in 5% fatty-acid-free dry milk in 500 mM NaCl, 20 mM Tris, and 0.05% Tween-20 (pH 7.4; TTBS) for 3 h at 4 °C. After blocking, the membranes were probed with antibodies against OMA1, NRF1, NDUFB6, Core II, DRP1, P616-DRP1 and P637-DRP1 proteins. After washing in TTBS, the membrane was incubated for 60 min with anti-rabbit or anti-mouse IgG peroxidase-conjugated antibodies and immunodetection was performed with enhanced chemiluminescence (ECL) (Euroclone, Paignton, UK) using ChemiDoc imaging system (BioRad, Milan, Italy). Densitometric analysis was performed by the Image Lab software (BioRad, Milan, Italy).

### 4.8. Data Analysis

The data presented in Figure 1, Figure 2, Figure 3, Figure 4, Figure 5 and Figure 6 are means ± SEM (standard error of the mean). The data presented in Table 1 are means ± SD (standard deviation). Statistical difference was determined by the Student’s *t*-test. A *p*-value < 0.05 was considered as statistically significant (* *p* < 0.05; ** *p* < 0.01; *** *p* < 0.001). For principal component analysis (PCA), data preprocessing and numerical manipulations were performed essentially as described in References [90,91]. Missing data were replaced by the intra-group averages.

## 5. Conclusions

Cancer is one of the most common causes of death globally. Despite extensive research and considerable advances in cancer therapy, the basis of the disease remains poorly understood. Understanding the key signaling mechanisms that contribute to tumor cell malignancy may help to discover new drug targets. Cyclic AMP is a signaling molecule that regulates a variety of cellular processes, including metabolism, proliferation, differentiation, and cell death. The cAMP-dependent signaling pathway is of particular interest in ovarian cancer as many hormones that control female reproductive function utilize this second messenger. Mitochondrial alterations play a key role in the metabolic reprogramming of tumor cells. Interestingly, in high-grade serous ovarian carcinoma, mitochondrial translation and OXPHOS therapies have been proposed as targets for therapy [92]. It is widely reported that the cAMP/PKA signaling pathway regulates several molecular mechanisms involved in mitochondrial function, biogenesis, and dynamics. In this study, we analyzed two serous ovarian cancer cell lines, OC316 and OV56, which showed different levels of cellular cyclic AMP, and serous OC tissues which can be distinguished into a group with low and another one with high levels of cAMP. We were able to verify that different levels of cyclic AMP are associated with different mitochondrial phenotypes in serous OC. Higher level of cyclic AMP was associated with a greater activation of the cAMP/PKA/PCREB/PGC-1α, cAMP/PKA/DRP1 and cAMP/SIRT3/OPA1 pathways that might be, in turn, associated with an increase in mitochondrial biogenesis and mitochondrial fusion, traits that could define a greater resistance to apoptosis. These results offer new targets of study for personalized therapy that consider the molecular differences within the same type of tumor.

## Figures and Tables

**Table 1:** Descriptive mitochondrial parameters for mitochondrial bioenergetics, biogenesis and dynamics were analyzed in the two populations of OC derived from PCA analysis. H-OC, tissues with higher cAMP levels (*n* = 6 with two or three technical replicates for each sample); L-OC, tissues with lower cAMP levels (*n* = 5 with two or three technical replicates for each sample). Student’s *t* test was considered significant for *p* < 0.05. n.s., not significant.

	Parameter (Unit of Measurement)	*H-OC* *means ± SD*	*L-OC* *means ± SD*	*p*
	Age (years)	61.6 ± 12.4	59.12 ± 16.1	n.s.
**Mitochondrial** **bioenergetics**	cAMP (pmol/mg prot)	9.3 ± 3.8	2.9 ± 1.1	0.0055
	Complex I (nmol/min/mg prot)	10.8 ± 3.9	14.2 ± 9.3	n.s.
	Complex II+III (nmol/min/mg prot)	60.0 ±16.6	29.91 ± 6.4	0.0042
	Complex IV (nmol/min/mg prot)	15.5 ± 4.3	16.2 ± 6.9	n.s
	ATP hydrolase (nmol/min/mg prot)	66 ± 17.5	41.3 ±10.3	0.036
	Cytrate Synthase (nmol/min/mg prot)	7.9 ± 3.5	3.4 ± 1.2	0.021
	ATP content (pmol/mg prot)	489.5 ± 74.3	194.9 ± 21.1	0.000013
**Mitochondrial** **biogenesis**	NDUFB6 protein level (ADU)	0.24 ± 0.11	0.2 ± 0.07	n.s
	CORE II protein level (ADU)	0.34 ± 0.1	0.25 ± 0.09	n.s
	COX IV protein level (ADU)	1.0 ± 0.37	1.2 ± 0.3	n.s
	TFAM protein level (ADU)	1.2 ± 1.0	0.57 ± 0.09	n.s
	NRF1 protein level (ADU)	2.6 ± 1.2	1.4 ± 0.7	n.s
	PGC-1α protein level (ADU)	1.8 ± 0.9	0.56 ± 0.2	0.032
	Mitochondrial DNA copy number	494.2 ± 40.8	315.8 ± 42.9	0.0005
	Mitochondrial number	18.5 ± 2.0	16.6 ± 1.8	0.044
**Mitochondrial** **dynamics**	SIRT3 protein level (ADU)	1.6 ± 0.4	1.2 ± 0.08	0.04
	OPA1 protein level (ADU)	10.5 ± 2.3	4.9 ± 3.2	0.009
	L-OPA1/S-OPA1	45.6 ± 3.7	38.4 ± 3.2	0.007
	DRP1 protein level (ADU)	1.5 ± 0.3	1.0 ± 0.3	0.002
	P616-DRP1/DRP1	1.4 ± 0.3	5.2 ± 1.5	0.0001
	PHB2 protein level (ADU)	5.9 ± 2.1	3.5 ± 1.9	n.s.
	OMA1 protein level (ADU)	0.8 ± 0.1	1.5 ± 0.4	0.002
	Mitochondrial max length (nm)	605.3 ± 100.8	489.9 ± 15.0	0.035

## Data Availability

The additional data supporting the manuscript are available from the corresponding author upon request.

## Supplementary Materials

The following supporting information can be downloaded at: https://www.mdpi.com/article/10.3390/ijms262110474/s1.

## Abbreviations

The following abbreviations are used in this manuscript:

BRCA1	Breast Cancer gene 1
BRCA2	Breast Cancer gene 2
cAMP	Cyclic adenosine monophosphate
COREII	Core subunit 2
COXIV	Cytochrome c oxidase subunit 4
CREB	cAMP response element-binding protein
DAPI	Diamidino-2-phenylinole
DRP1	Dynamin-related protein 1
HGSOC	High-grade serous ovarian cancer
MAGE-A11	Melanoma-associated antigen 11
MFN2	Mitofusin 2
NDUFA9	NADH:ubiquinone oxidoreductase subunit A9
NDUFB6	NADH:Ubiquinone Oxidoreductase Subunit B6
NDUFS4	NADH:Ubiquinone Oxidoreductase Subunit S4
NRF1	Nuclear respiratory factor 1
OC	Ovarian cancer
OMA1	Overlapping proteolytic activity with m-AAA protease 1
OPA1	Optic atrophy 1
PAGE	Polyacrylamide gel electrophoresis
PCA	Principal component analysis
PCG-1α	Peroxisome proliferator-activated receptor gamma coactivator 1-alpha
PHB2	Prohibitin 2
PKA	Protein kinase A
PMSF	Phenylmethylsulfonyl fluoride
SDS	Sodium dodecyl sulfate
SIRT3	NAD-dependent deacetylase sirtuin-3
TFAM	Mitochondrial transcription factor A
