# Supplementary material for: Cellular cAMP Content and Mitochondrial Profile Define Different Subtypes of Ovarian Cancer Cells
Source: Int J Mol Sci. 2025 Oct 28;26(21):10474. doi: 10.3390/ijms262110474 (PMC12607473; doi:10.3390/ijms262110474)
Supplement: Supplementary file 1 [file ijms-26-10474-s001.zip › ijms-3843854-supplementary.pdf]

MTT Test

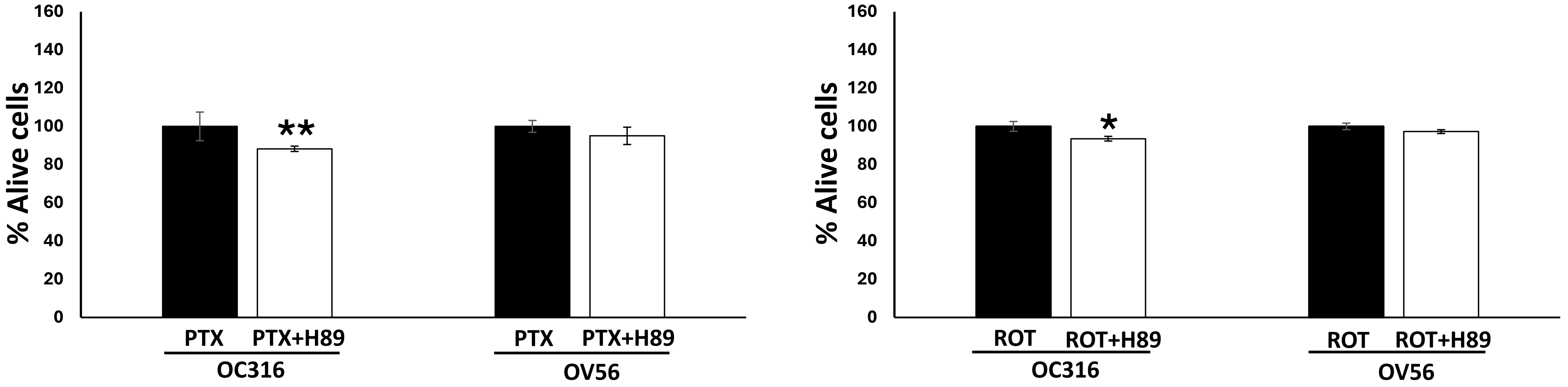

***Figure S1**. Analysis of viable cells after pre incubation with H89 followed by apoptosis induction treatments with paclitaxel or rotenone.* Cells were seeded in 96-well plates. After 24 hours, H89 (48 nm) was added to the medium for further 24 hours and then paclitaxel or rotenone was added. Following treatment, the culture medium was removed, and a 0.25 mg/mL MTT solution (Sigma Aldrich, Saint Louis, MO, USA) was added to each well. Plates were incubated for 2 hours at 37°C. The MTT solution was then discarded, and the resulting formazan crystals were solubilized using isopropanol (Sigma Aldrich). Absorbance was measured at 590 nm using a spectrophotometer. Data in the histograms represent cell viability in the presence of rotenone or paclitaxel after 24 pre-incubation with H89 (48 nm). Data are expressed as percentage of the mean values ± SEM of three independent determinations derived from different stocks of the same cell lines. *p<0.05; **p<0.01 (Student's *t*-test). All experimental conditions were performed in triplicate.

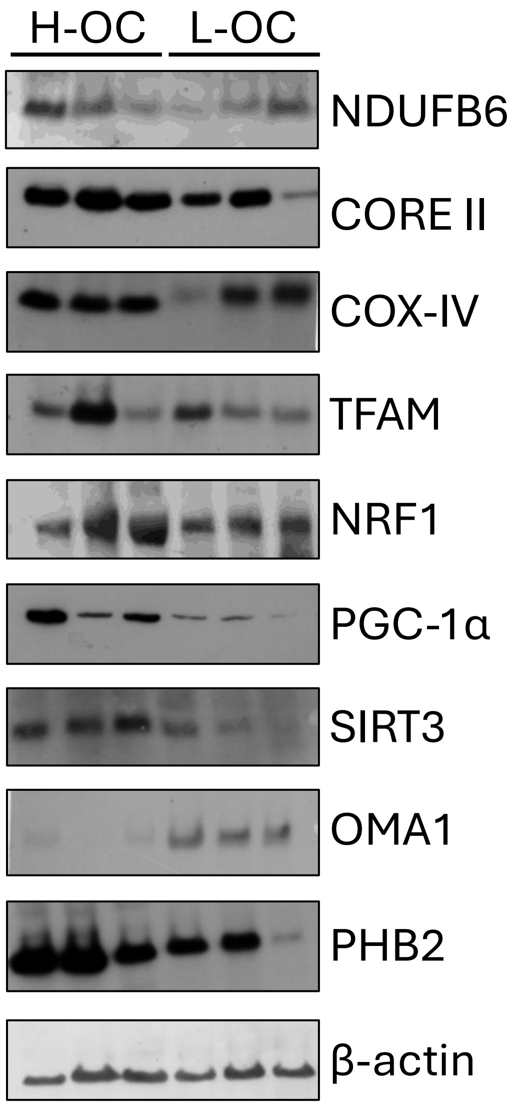

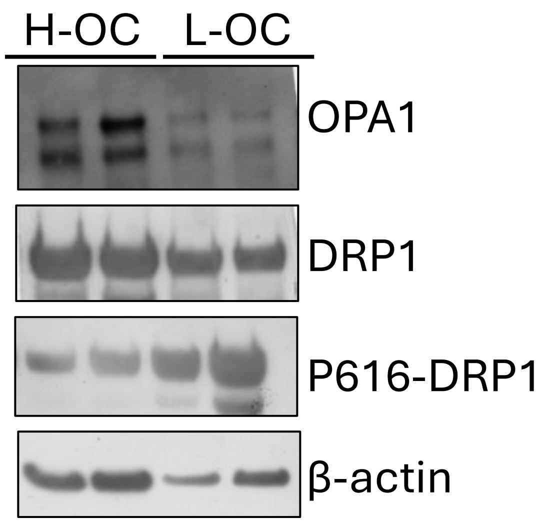

Figure S2. Representative images of western blotting analysis in OC tissues with higher cAMP level (H-OC) and with lower cAMP level (L-OC). The sample tissues were homogenized and centrifuged at 600xg; the resulting supernatant was sonicated and utilized for western blotting analysis. Proteins were loaded on 8% SDS-polyacrylamide gel electrophoresis (PAGE). After separation, the proteins were transferred on nitrocellulose membranes and immunoblotted with the antibodies described in the figures. The values of the densitometric arbitrary units (ADU) ± standard deviation (SD) are shown in Table 1.

Supplemental Table S1.

Cell Lines used in the study from https://www.cellosaurus.org/:

- OVCAR-8 Homo sapiens (Human) CVCL_1629
- SK-OV-3 Homo sapiens (Human) CVCL_0532
- OC316 Homo sapiens (Human) CVCL_1618
- OV56 Homo sapiens (Human) CVCL_2673
- IGROV-1 Homo sapiens (Human) CVCL_1304

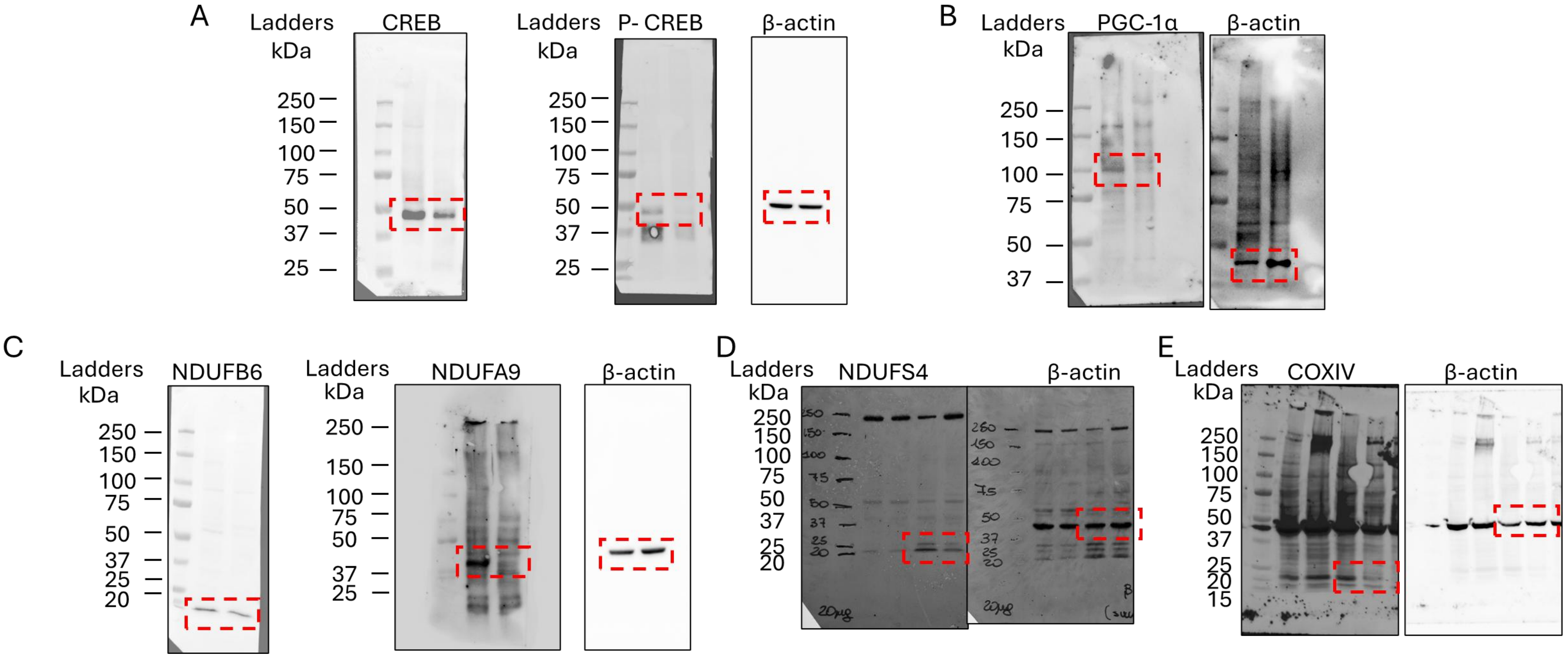

Figure S3. (A) Full scan of western blotting analysis of CREB and P-CREB showed in Figure 3A of the manuscript. The ladders and western blotting derived from composite image between colorimetric and chemiluminescence acquisitions using ChemiDoc instrument (Bio-Rad) and Image Lab software (Bio-Rad). For β-actin, the same membrane was re-probed. (B) Full scan of western blotting analysis of PGC1-α showed in Figure 3B of the manuscript. The ladders and western blotting derived from composite image between colorimetric and chemiluminescence acquisitions using ChemiDoc instrument (Bio-Rad) and Image Lab software (Bio-Rad). For β-actin, the same membrane was re-probed. (C) Full scan of western blotting analysis of NDUFB6 and NDUFBA9 showed in Figure 3C of the manuscript. The ladders and western blotting derived from composite image between colorimetric and chemiluminescence acquisitions using ChemiDoc instrument (Bio-Rad) and Image Lab software (Bio-Rad). For β-actin, the same membrane was re-probed. (D) Full scan of western blotting analysis of NDUFS4 showed in Figure 3D of the manuscript obtained by detection on Hyperfilm ECL (GE Haelthcare-Amersham) with ladders manually marked. For β-actin, the same membrane was re-probed. (E) Full scan of western blotting analysis of COXIV showed in Figure 3E of the manuscript obtained by detection on Hyperfilm ECL (GE Haelthcare-Amersham) with ladders manually marked. For β-actin, the same membrane was re-probed.

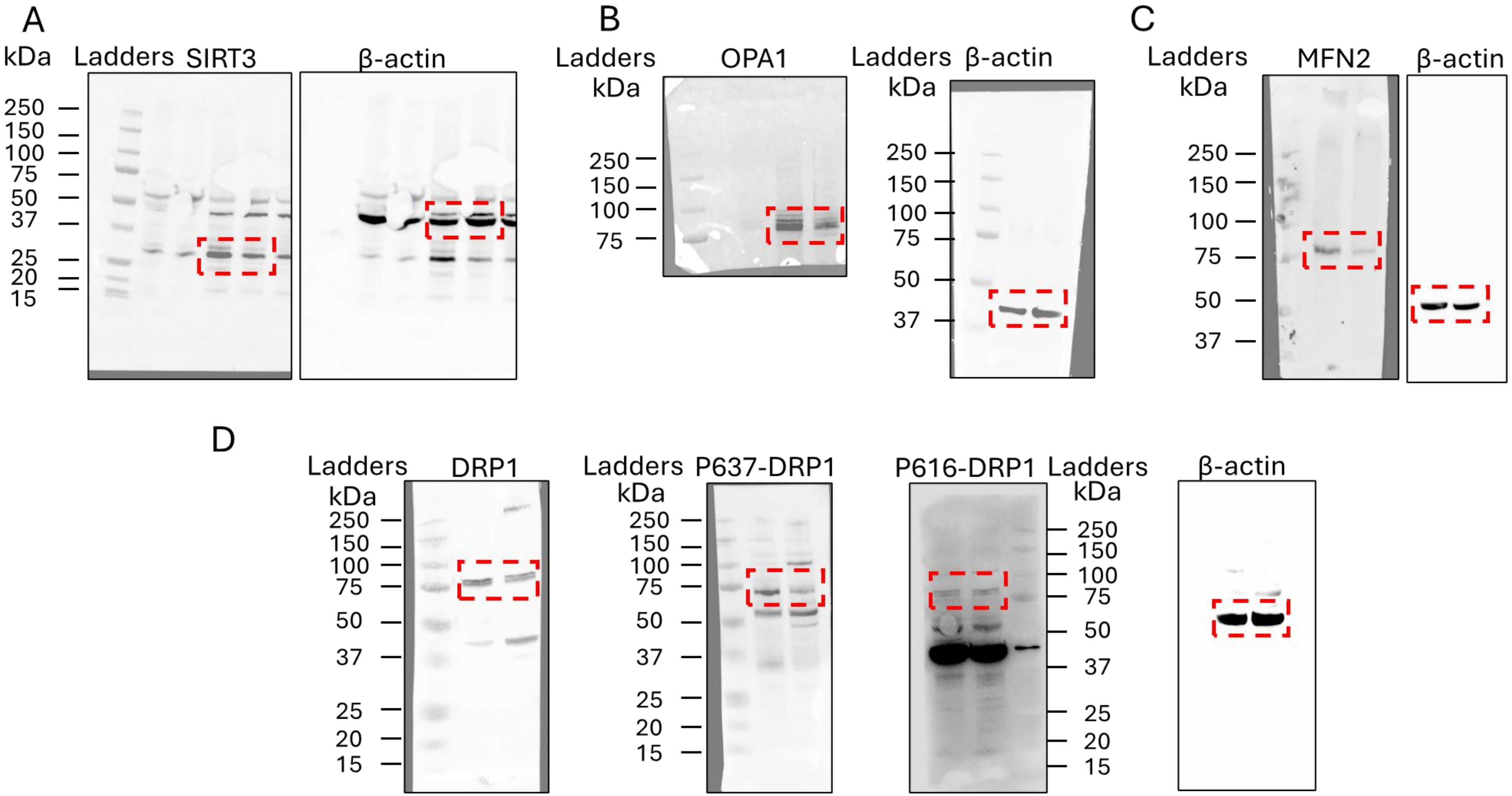

Figure S4. (A) Full scan of western blotting analysis of SIRT3 showed in Figure 6A of the manuscript. The ladders and western blotting derived from composite image between colorimetric and chemiluminescence acquisitions using ChemiDoc instrument (Bio-Rad) and Image Lab software (Bio-Rad). For β-actin, the same membrane was re-probed. (B) Full scan of western blotting analysis of OPA1 showed in Figure 6B of the manuscript. For β-actin, the same samples were re-loaded and membrane was probed with antibody against β-actin. The ladders and western blotting derived from composite image between colorimetric and chemiluminescence acquisitions using ChemiDoc instrument (Bio-Rad) and Image Lab software (Bio-Rad). (C) Full scan of western blotting analysis of MFN2 showed in Figure 6C of the manuscript. The ladders and western blotting derived from composite image between colorimetric and chemiluminescence acquisitions using ChemiDoc instrument (Bio-Rad) and Image Lab software (Bio-Rad). For β-actin, the same membrane was re-probed. (D) Full scan of western blotting analysis od DRP1, P637-DRP1 and P616-DRP1 showed in Figure 6D of the manuscript. The ladders and western blotting derived from composite image between colorimetric and chemiluminescence acquisitions using ChemiDoc instrument (Bio-Rad) and Image Lab software (Bio-Rad). For β-actin, the same membrane was re-probed.

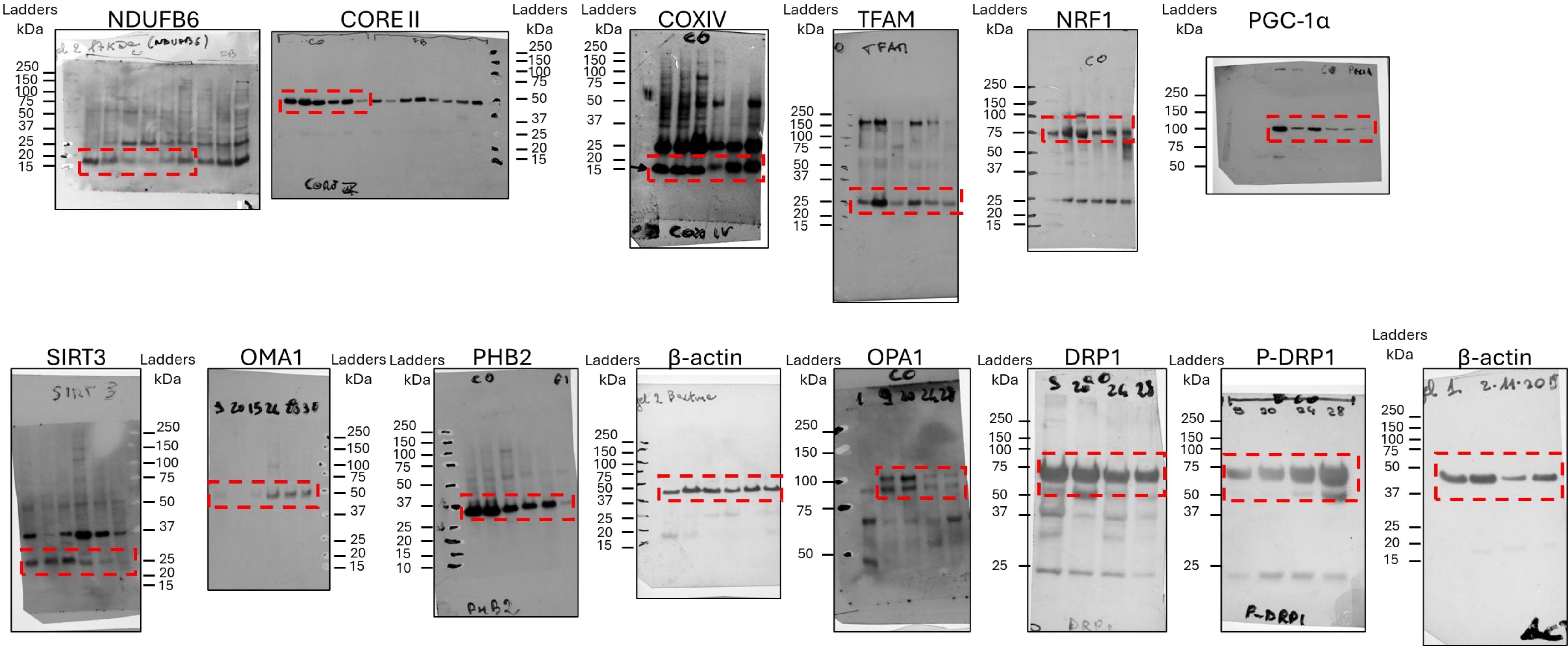

Figure S5. Full scan of western blotting analysis shown in Figure S2 of the manuscript obtained by detection on Hyperfilm ECL (GE Haelthcare-Amersham) with ladders manually marked.
